# Effect of smoking status on neuronal responses to graphic cigarette warning labels

**DOI:** 10.1371/journal.pone.0201360

**Published:** 2018-09-20

**Authors:** Tobias Rüther, Yannick Schultz, Christina Wirth, Agnieszka Chrobok, Andrea Rabenstein, Daniel Keeser, Birgit Ertl-Wagner, Oliver Pogarell, Susanne Karch

**Affiliations:** 1 Department of Psychiatry und Psychotherapy, Ludwig Maximilian University, Munich, Germany; 2 Institute of Clinical Radiology, Ludwig Maximilian University, Munich, Germany; University of Montreal, CANADA

## Abstract

**Background:**

Smoking is responsible for a large proportion of cancer, respiratory and cardiovascular deaths. Nevertheless the health risks of smoking are still underestimated in many smokers. The present study aimed to examine neurobiological responses to graphical warnings on cigarette packings in non-smokers and patients with tobacco dependence.

**Methods:**

Twenty non-smokers and twenty-four patients with tobacco dependence participated in a functional MRI study during that pictures of different categories were presented ((a) EU-warning pictures, (b) text-only warnings, (c) neutral pictures with short information). Patients contributed twice in the experiment (after 10 hours nicotine withdrawal / about 5 minutes after nicotine consumption).

**Results:**

Smokers during withdrawal demonstrated increased neuronal responses predominantly in subcortical, temporal and frontal brain regions that are associated with emotional and cognitive processes during the presentation of graphical warnings compared to neutral pictures. In smokers after smoking and non-smokers, the differences between graphical warnings and neutral pictures were increased compared to smokers during withdrawal. The comparison of the graphical warnings with text-only labels demonstrated the importance of affective brain regions especially in smokers after smoking and in non-smokers. During withdrawal, the neural responses associated with graphical warnings and text-only labels differed only marginally.

**Discussion and conclusion:**

The results suggest that emotional and cognitive reactions to graphical warnings are predominantly seen in smokers after smoking and in non-smokers. The impact of these pictures during withdrawal seems to be less pronounced; in this case, more unspecific processes seem to be important, including the projection of sensory signals to the cerebral cortex.

## Introduction

Smoking is responsible for a large proportion of cancer, respiratory and cardiovascular deaths and is believed to be the single most important cause of death in Europe [1,2,3]. In 2015, the World Health Organization estimated a number of 6 million deaths attributable to smoking, and a number of 600,000 deaths of nonsmokers exposed to second-hand tobacco smoke (WHO 2015, http://www.who.int/mediacentre/factsheets/fs339/en/). In all likelihood most people are aware of the general harmfulness of smoking but many smokers still underestimate the risks of smoking and the associated range of illnesses for themselves and others [4](WHO 2011).

One strategy for prevention is warning labels on cigarette packaging whose implementation is progressing rapidly. While text-only messages are prevalent in most countries, many countries are now increasing warning signs, and a growing number has introduced graphic warning labels (GWL) [5]. Whereas in 2012 55 countries/jurisdictions had implemented picture warning requirements, their number has risen to 77 by September 2014. This means that picture warnings now reach 49% of the world’s population [5]. Since May 2016, all 28 EU countries require GWLs to cover 65% of packages front and back [6].

In literature, the prevailing view is that GWLs are more effective than text-only warnings in enhancing risk perception and health knowledge [7,8]. One important advantage is that pictorial warning labels have demonstrated to elicit enhanced emotional responses compared to text-only warnings [9]. Emotional responses to GWLs were negative including e.g. fear, disgust [10,11].

In particular, large and prominent GWLs have been proved to be effective [7,12]. It has been shown that large GWLs are a significant source of health information for both smokers and non-smokers [13]. Exposure to GWLs discourage smoking initiation [14,15], increasing health knowledge, perception and awareness of risks associated with smoking [14,15,16,17,18,19]. They reduce the appeal of cigarette packets [14], restrict intentions to stop smoking [17], encourage cessation [14,15,20,21,22], prevent relapse [23] and increase the use of quit lines [24]. It is assumed that GWLs provoke high emotions which support the implementation of behavioural change like quitting smoking [25].

While the beneficial effects of GWLs on behaviour correlates are well documented, research on the neurobiological basis for the efficacy of GWLs is still rare. In the last few years, neuroimaging studies focused on neurobiological correlates of smoking cue-related information. The neurobiology of smoking cue reactivity has been summarised by a meta-analysis indicating that the most important areas in smoking cue reactivity are the precuneus, the posterior and anterior cingulate cortex (ACC), the dorsal and medial prefrontal cortex (DLPFC, MPFC), the superior and inferior parietal lobules, the insula and the dorsal striatum [26]. GWLs appear to be the counterpart of smoking cues but little is known about their neurobiological effect so far. Newman-Norlund and colleagues (2014) indicated that pictorial warnings activate large-scale neural networks including amygdala, insula, visual association cortex, hippocampus, fusiform gyrus, precentral gyrus, supplementary motor area, pars triangularis, pars opercularis, pars orbitalis and fusiform gyrus [27]. Another study demonstrated an association between frontoinsular neural activity and craving reduction in response to GWLs [28]. The amygdala was most robustly activated by warnings that included personal suffering from smoking-related consequences followed by warnings, including graphic representation of physical consequences of smoking and symbolic representations of risk [27].

Green and colleagues (2016) revealed increased neuronal responses especially in the medial prefrontal cortex, the amygdala, the medial temporal lobe, and the occipital cortex during the presentation of GWLs in young adult smokers. There were no significant differences in response to warnings on branded versus plain cigarette packages. The self-reported motivation to quit smoking was significantly higher after viewing GWLs compared to control pictures [29]. Wang and colleagues (2015) demonstrated that GWLs causing high emotional reactions were associated with increased responses e.g. in the amygdala, the hippocampus, the inferior frontal gyri and the insula. The neurobiological variations were accompanied by a greater reduction in the craving to smoke [25]. Overall brain regions activated during the presentation of GWLs are assumed to be involved in cognitive and affective decision-making and memory formation [25,27,29] as well as visual information processing. Particularly the amygdala is associated with emotion processing and emotional evaluation of sensory stimuli [30,31]. The amygdala response is associated with quitting smoking [32].

The aim of the present study was to investigate the neuronal responses to the new German GWLs in non-smokers and smokers, especially taking into account the smoking status of smokers (smokers after deprivation versus smoker after smoking). Withdrawal can lead to withdrawal symptoms including craving and anxiety within several hours. Diagnostic criteria of the nicotine withdrawal syndrome are irritability, decreased frustration tolerance, anger, aggression, attention deficit, restlessness, dysphoric mood [33]. Avoidance of the negative state produced by nicotine withdrawal represents a motivational component that promotes continued tobacco use and relapse after smoking cessation. With the modest success rate of currently available smoking cessation therapies, understanding mechanisms involved in the nicotine withdrawal syndrome are crucial for developing successful treatments [34]. Overall, the available literature indicates that the nicotine withdrawal syndrome is complex, and involves a range of neurobiological mechanisms [34].

While undergoing functional magnetic resonance imaging (fMRI), the new GWLs, old text-only warnings and neutral pictures were presented to the participants.

We hypothesised that especially regions associated with emotion processing [e.g. the amygdala [35,36]], craving suppression [including the DLPFC [37] and the insula [38]] would show significant responses during the presentation of the new German GWLs. These responses are supposed to be increased during the presentation of the new GWLs compared to the text-only warnings and neutral pictures. In addition, we hypothesised that neural responses associated with new GWLs would be increased in smokers compared to healthy subjects because of the stronger personal relevance of the information for smokers.

## Materials and methods

### Sample

24 smokers (male: n = 12; female: n = 12) and 20 non-smokers (male: n = 10; female: n = 10) aged between 23 and 53 years were recruited via online advertisements and were screened for eligibility in a telephone interview. One inclusion criterion was the age between 18 and 65; only smokers with a Fagerström score of > 3 were included. Participants who were not able to maintain the smoking deprivation of 10 hours, who reported a current/past neurological or psychiatric disorder and who met standard fMRI exclusion criteria (e.g. pregnancy, metal, claustrophobia) were not included in the study. Written informed consent was obtained from each participant after procedures had been fully explained. The consent procedures were approved by the ethics committee of the Ludwig-Maximilians-University.

Smokers and non-smokers did not differ significantly in all assessed, smoking-unrelated characteristics (age, sex, level of education, verbal intelligence, personality, impulsiveness, depressive symptoms). Please see Table 1 for further information.

**Table 1:** Characteristics of the sample.

		Smokers (*n* = 24)	Non-smokers (*n* = 20)	Total (*n* = 44)
Age	Mean (SD)	30.29 (7.64)	30.60 (6.44)	30.43 (7.04)
	Range	23–53	24–52	23–53
Sex	Male	12	10	22
	Female	12	10	22
IQ	Mean (SD)	112.08 (10.03)	114.37 (10.38)	113.09 (10.13)
Years of general education	0–11	5 (20.8%)	3 (15.0%)	8 (16.3%)
	12–13	19 (79.2%)	16 (80.0%)	35 (71.4%)
	Missing	0	1 (5.0%)	1 (12.3%)
Cigarettes/day	Mean (SD)	20.83 (6.33)	0*	
Fagerström score	Mean (SD)	6.33 (1.52)	0*	
Length of regular Tobacco consumption	Mean (SD)	14.04 (6.27)	0*	
	Range (years)	5–25		
CO level in ppm	After withdrawal	9.00 (4.21)*		5.61 (4.88)
	After consumption	22.00 (6.88)*		14.59 (11.59)
Cotinine level	After withdrawal			
	After consumption			
Depressive symptoms in BDI	Mean (SD)	4.65 (4.19)	2.58 (2.76)	3.73 (3.74)
Impulsiveness in BIS	Attentional Impulsiveness	22.00 (4.01)	23.84 (3.70)	22.81 (3.99)
	Motor Impulsiveness	22.96 (4.54)	23.63 (4.31)	23.26 (4.40)
	Non-planning Impulsiveness	22.29 (3.39)	33.32 (4.24)	22.26 (3.74)

* denotes significant group differences (p < .05).

Abbreviation: SD: standard deviation

### Assessment of behavioural and smoking related data

Several questionnaires were used for the assessment of behavioural and smoking related data. The smoking urge was assessed by the German version of the Questionnaire of Smoking Urges (QSU-G [39], aggression by the Aggression Questionnaire (AQ, [40,41]), impulsiveness by the Barratt Impulsiveness Scale (BIS-11 [42], intelligence by a German verbal intelligence test (Wortschatztest (WST) [43]), depressive symptoms by the Beck Depression Inventory (BDI-II [44]) and nicotine dependence by The Fagerström Test of Nicotine Dependence (FTND [45,46]). Further assessed information included age, sex, level of education and number of cigarettes smoked per day, and is summarised in Table 1.

### Procedure of graphic warning label task

The study was approved by the ethics committee of the Medical Department of the Ludwig-Maximilians-University Munich. FMRI measurements took place at the Institute of Clinical Radiology, Ludwig-Maximilians-University Munich. At the first session, participants provided written informed consent. The smoking status was confirmed by carbon monoxide (CO) in expired air by a Bedfont Smokerlyzer and by salivary cotinine levels. A basic questionnaire (demographics, smoking behaviour, BIS-11, WST, NEO), QSU and AQ were completed and the fMRI session was started.

Non-smokers and smokers participated at the fMRI sessions twice on separate days: for smokers (a) after 10 hours nicotine withdrawal; (b) directly (about 5 minutes) after nicotine consumption. The sequence of measurements (a-b vs. b-a) was counterbalanced between subjects.

**Fig 1:**
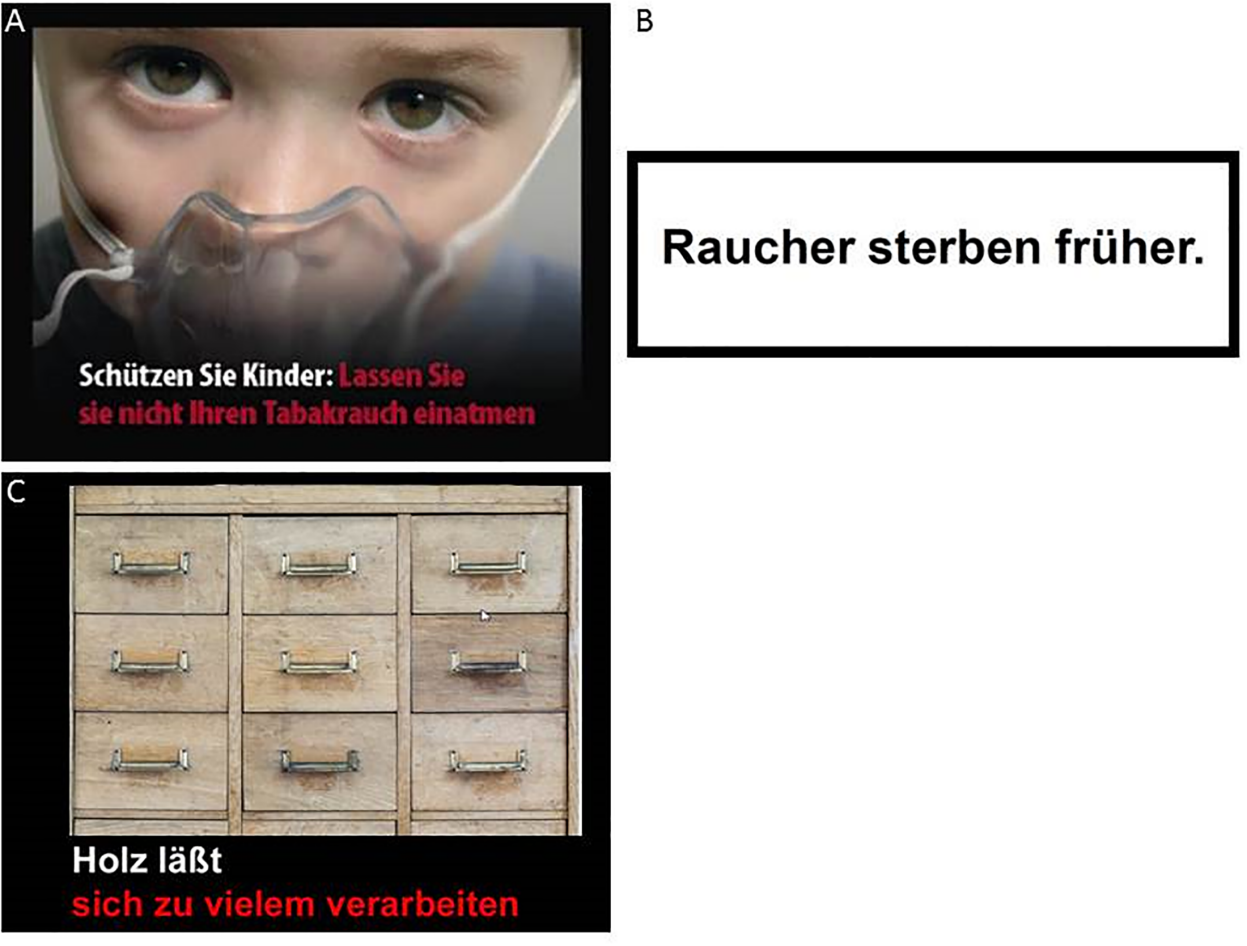
Example pictures of the three stimulus categories A) graphical warning notices (translation: Protect children, don’t let them breath in your tobacco smoke); B) warning notices (translation: Smokers die earlier); C) neutral pictures (translation: Wood can be made into anything).

There were three categories of visual stimulation: (1) 32 warning pictures with the EU warning notices (date: 10.09.2013), (2) 16 warning notices (old text-only warning labels), (3) 32 neutral pictures with short information. The design was kept according to the official EU warning notices. Fig 1 shows an example of the three stimuli categories.

In order to determine the neutrality of the neutral pictures the emotional valence and arousal has been rated in a sample of 21 subjects using the Self Assessment Manikin scores. The subject can select any of the 5 figures comprising each scale, or between any two figures, which results in a 9-point rating scale for each dimension. Ratings are scored such that 9 represents a high rating on each dimension (i.e., high pleasure, high arousal), and 1 represents a low rating on each dimension (i.e., low pleasure, low arousal). The mean *valence* score for the neutral pictures was m = 5.47 (standard deviation: 0.31; minimum: 4.63; maximum: 5.97). The mean *arousal* for the neutral pictures was m = 2.42 (standard deviation: 0.44; minimum: 1.34; maximum: 3.25).

The pictures were presented for 6 seconds each. The picture sequences differed between the first and the second fMRI session in order to prevent that subjects know which picture will be presented next. The same picture sequences were used for smokers and non-smokers. The subjects were instructed to view the pictures on the screen.

We especially focused on neuronal alterations that are linked to motivational processes within the emotion network. As we supposed that the combination of cognitive tasks with emotional information could be used by some participants as a distraction strategy in order to prevent dealing with the emotional content of the pictures and/or to control emotional responses, we did not include any behavioural task.

After the fMRI session, participants were asked again to complete the QSU and the AQ.

### MRI data acquisition and analysis

FMRI-imaging was performed in a 3 Tesla Philips scanner with echoplanar capability. A three-dimensional MPRAGE data set (T1-weighted) was acquired for each subject for anatomical referencing. For functional BOLD imaging during the presentation of pictures a T2* weighted EPI sequence was acquired in the same position as the anatomical images (repetition time (TR): 2000 ms; echo time (TE): 30 ms; 36 axial slices; matrix size: 1,65 x 1,65; slice thickness: 3 mm).

The post-processing and analysis of the fMRI data was carried out by the BrainVoyager software package (Brain Innovation, Maastricht, Netherlands). The first 4 images were excluded from any further analysis due to relaxation time effects. The preprocessing of the functional data included high-pass filtering (cut-off: three cycles in time course) to low frequency signal drift inherent in echo planar imaging, a slice scan time correction, spatial smoothing (Gaussian filter with FWHM 8.0 mm), and a 3D motion correction. In addition, the functional images were transferred to a standard Talairach brain. Significant BOLD activity was determined by a cross correlation of MR image pixel intensity with an expected hemodynamic response function. Voxelwise t-tests were used to identify those brain areas where the signal change was significantly different between the different experimental conditions (*new GWLs*, *text-only labels*, *neutral* pictures). For each participant the conditions *new GWL*, *text-only label* and *neutral pictures* were calculated as regressors.

The results of non-smokers were compared to those of smokers after nicotine consumption as well as smokers during withdrawal. The data of all smokers and non-smokers were calculated in the same GLM.

### Statistical analysis

Nominal data (demographics) were compared by Chi-square tests and continuous data by one-way ANOVAs. Paired t-tests were conducted to compare the impact of the fMRI session on smoking urge (craving), intention to smoke and aggression. All tests of significance used an alpha level of 0.05 and were reported as 2 tailed. Statistical analyses were performed with the software SPSS version 23.0 for Windows.

## Results

### Functional MRI results

#### New GWLs versus neutral pictures

**Fig 2:**
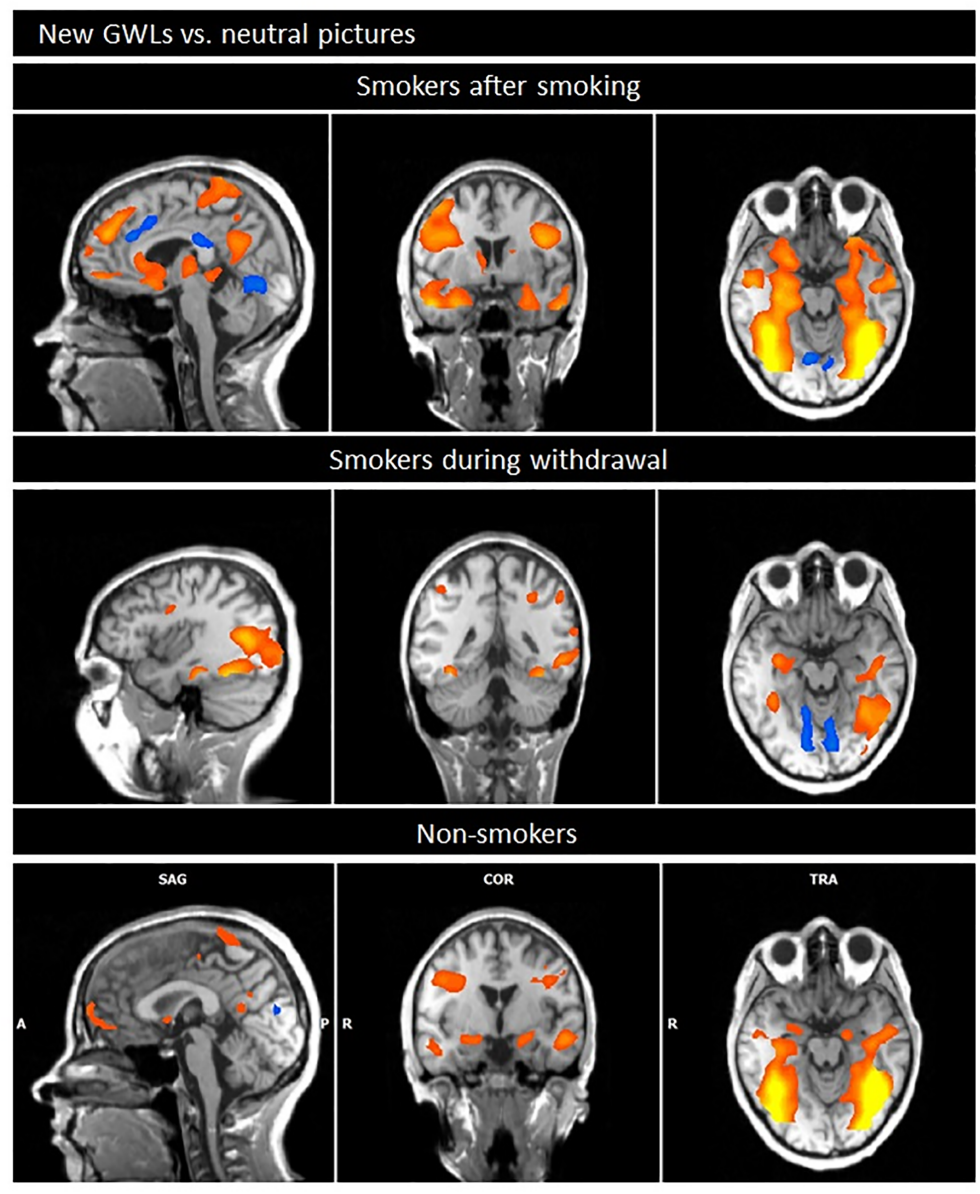
Comparison of neuronal responses during the presentation of the new GWLs minus neutral pictures.

In the comparison of the new GWLs with neutral pictures, *smokers after smoking* showed increased BOLD responses especially in subcortical, temporal and frontal regions including amygdala/parahippocampal gyrus, superior and middle temporal gyrus, e.g. thalamus/globus pallidus/caudate, insula, hippocampus, middle/superior/inferior frontal gyrus, anterior cingulate cortex, precentral/postcentral gyrus. In addition, enhanced responses were shown e.g. in the superior parietal gyrus, postcentral and precentral gyrus, precuneus, superior/middle temporal gyrus. Decreased responses during the new GWLs compared to the neutral cues were small (e.g. middle/superior frontal gyrus) (see Table 2, Fig 2).

**Table 2:** Smokers after smoking: Comparison of new GWLs and neutral cues (Fixed effects analysis q(FDR) < 0.01, T: 2.92–8.0; p < 0.003547).

			Center of Mass			Size	T-value	
Brain region	Site	BA	x	y	z		Ø	Max
**A: Smokers after smoking: new GWLs > neutral cues**								
**Subcortical**								
Insula Transverse temporal gyrus / Putamen	R	13/41	31	-25	11	1671	3.49	4.70
Insula / Inferior frontal gyrus	R	13/47	31	29	-5	5206	4.37	6.58
Insula / Transverse temporal gyrus	R	13	32	-24	11	1947	3.48	4.72
Caudate / Anterior cingulate	L/R	25	0	14	2	11133	3.77	5.96
Caudate / Putamen / Globus pallidus	L		-11	7	8	1380	3.42	4.57
Thalamus / Caudate	R		6	0	8	3438	3.74	5.51
Thalamus	L		-9	-15	9	3414	3.33	4.58
Thalamus	R		7	-9	12	2254	3.55	5.00
Hippocampus Globus pallidus / Putamen / Caudate	R		30	-13	-9	47267	4.22	7.38
Hippocampus Caudate / Parahippocampal gyrus	L	20/21/36	-36	-19	-12	37026	4.27	11.56
Caudate	L		-7	15	4	5146	4.62	5.35
**Frontal Lobe**								
Posterior cingulate gyrus Precuneus	R	7/23/30/31	2	-53	26	12738	4.27	7.43
Medial / superior frontal gyrus	R	9	1	46	27	1851	4.30	6.61
Inferior / middle frontal gyrus Precentral gyrus	R	6/9	40	8	29	46400	4.39	8.15
Inferior / middle frontal gyrus	R	11/47	32	28	-6	5909	4.32	6.58
Inferior / middle frontal gyrus	L	9/44-46	-49	18	18	3362	4.08	6.06
Medial frontal gyrus Anterior cingulate	R	6/9/32	10	41	30	19524	4.51	7.08
Medial / superior frontal gyrus	L	6/8/9	-8	44	33	9707	3.91	6.42
Pre-/postcentral gyrus Middle frontal gyrus	L	4/6	-39	-12	42	23214	3.71	6.33
**Parietal Lobe**								
Superior and inferior parietal lobule Precuneus	L	7/40	-30	-52	52	6959	3.97	6.33
Superior parietal lobule / Precuneus	R	7	25	-61	51	5012	4.56	7.83
Superior parietal lobule / Precuneus	L	7/19	-26	-71	45	930	3.49	4.56
Pre-/postcentral gyrus	R	3/4	20	-30	63	15959	3.92	6.55
Postcentral gyrus Inferior parietal lobule	R	2/40	48	-27	41	5749	3.92	5.75
Superior / paracentral parietal lobule Precuneus / Postcentral gyrus	L	5/7	-11	-51	65	4057	3.65	4.91
**Temporal Lobe**								
Superior /middle temporal gyrus	L	21/22/42	-64	-26	7	387	3.25	4.03
Middle / superior temporal gyrus	R	19/22	34	-59	9	85216	5.5	12.5
Middle / superior temporal gyrus	L	21/22	-47	-45	2	12509	4.87	11.15
Transverse temporal gyrus Superior temporal gyrus	L	21/22/42	-63	-19	8	1010	3.33	4.20
Middle / inferior temporal gyrus	L	20/21	-53	-6	-17	4408	4.05	5.87
**Occipital Lobe**								
Middle occipital / Middle temporal gyrus	R	19/37	36	-64	10	67644	5.87	12.50
Middle occipital / Middle temporal gyrus	L	19/37	-37	-61	4	63538	5.93	12.78
**B: Smokers after smoking: new GWLs < neutral cues**								
**Frontal Lobe**								
Cingulate gyrus / Medial frontal gyrus	L	6/8/9/32	-6	21	35	2325	3.36	4.58
(Posterior) cingulate gyrus	L	23/31	-5	-26	26	939	3.23	3.80
Middle / superior frontal gyrus	L	9/10	-32	41	28	4906	4.00	6.03
**Cerebellum**								
Declive Culmen / Declive of Vermis	R/L		-1	-67	-9	4562	3.44	4.40

Abbreviations: R: right, L: left; Centre of mass: Talairach Coordinate; Size: cluster size; Ø: mean value

*During withdrawal smokers* demonstrated enhanced neuronal responses while the new GWLs were presented, compared to the neutral cues predominantly in the amygdala/hippocampus, middle/inferior temporal/occipital gyrus, the inferior parietal gyrus/postcentral gyrus and the inferior/middle frontal region. The responses in the caudate/cuneus as well as the superior/middle frontal gyrus were decreased during the new GWLs compared to the neutral condition (Table 3, Fig 2).

**Table 3:** Smokers during withdrawal: Comparison of new GWLs and neutral cues (Fixed effects analysis q(FDR) < 0.01, T: 3.35–8.0; p <0.000825).

			Center of Mass			Size	T-value	
Brain region	Site	BA	x	y	z		Ø	Max
**A: Smokers during withdrawal: new GWLs > neutral cues**								
**Frontal Lobe**								
Inferior frontal gyrus	L	13/45/47	-48	24	3	718	3.58	4.10
Precentral gyrus Inferior / middle frontal gyrus	L	6/9	-44	2	31	842	3.72	4.52
**Parietal Lobe**								
Inferior Parietal Lobule / Postcentral Gyrus	L	1/2/40	-55	-30	39	1158	3.71	4.43
Inferior Parietal Lobule, Postcentral Gyrus / Supramarginal gyrus	R	1/2/40	53	-30	36	1936	3.75	4.58
**Subcortical / Temporal lobe / Occipital lobe**								
Amygdala / Hippocampus	R		30	-13	-14	1374	3.79	5.21
Fusiform gyrus	R	20/37	36	-43	-17	1067	4.11	5.71
Middle / inferior temporal gyrus Middle occipital gyrus	R	19/37	44	-64	3	11938	4.70	7.41
Middle / inferior temporal gyrus Middle occipital gyrus	L	19/37	-45	-58	-2	26436	5.01	8.59
**B: Smokers during withdrawal: new GWLs < neutral cues**								
**Frontal Lobe**								
(Anterior) cingulate gyrus Medial frontal gyrus	R	9/32	19	29	24	5288	3.80	4.89
Superior / middle frontal gyrus	R	10	19	56	9	1291	4.2	5.67
Superior / middle frontal gyrus	L	10	-21	55	12	1537	3.83	4.80
**Subcortical / Occipital Lobe**								
Lingual gyrus / Cuneus	R	18/30	1	-74	2	18249	3.93	5.73
Caudate	R		22	-12	28	2389	3.86	4.89
Caudate	L		-18	20	17	1861	3.78	4.71

Abbreviations: R: right, L: left; Centre of mass: Talairach Coordinate; Size: cluster size; Ø: mean value

*Non-smokers* showed enhanced BOLD responses during the presentation of new GWLs compared to neutral cues e.g. in amygdala/parahippocampal gyrus/hippocampus, middle occipital/temporal gyrus, anterior cingulate cortex, superior/middle and inferior frontal gyrus and inferior parietal cortex/postcentral gyrus. Decreased responses were small and predominantly located in the cuneus/lingual gyrus (Table 4, Fig 2).

**Table 4:** Non-smokers: Comparison of new GWLs and neutral cues (Fixed effects analysis q(FDR) < 0.01, T: 3.12–8.0, p < 0.001792).

			Center of Mass			Size	T-value	
Brain region	Site	BA	x	y	z		Ø	Max
**A: Non-smokers: new GWLs > neutral cues**								
**Subcortical**								
Caudate / Globus pallidus / Thalamus	R		8	4	4	1066	3.45	4.15
Amygdala Parahippocampal gyrus	L	28/34	-20	-7	-13	885	3.52	4.30
Amygdala Hippocampus / Parahippocampal gyrus	R	28/34	26	-9	-14	2073	3.53	4.90
Parahippocampal gyrus / Thalamus Culmen	R	27/30	14	-34	0	2118	3.75	5.04
**Frontal Lobe**								
Anterior cingulate Medial / superior frontal gyrus	L	10/32	-17	43	-2	3238	3.64	4.99
Anterior cingulate Medial / superior frontal gyrus	R	10/32	4	52	-1	834	3.45	4.08
Inferior / middle frontal gyrus Precentral gyrus	L	6/9	-40	7	26	7481	3.64	5.01
Precentral gyrus / Inferior frontal gyrus	R	6	36	-2	31	8823	3.63	4.95
Medial / superior frontal gyrus	L/R	10	-1	63	8	1845	3.50	4.28
**Parietal Lobe**								
Inferior parietal lobule / Postcentral gyrus	L	3/40	-34	-36	50	14011	3.60	4.78
Inferior parietal lobule / Postcentral gyrus	R	2/3/40	54	-25	29	2709	3.60	4.68
Inferior parietal lobule / Precuneus Postcentral gyrus Superior / paracentral lobule	R	5/7/40	27	-40	45	21264	3.78	6.21
Postcentral gyrus / Precuneus	R	5/7	1	-46	66	2219	3.45	4.20
Precuneus / Cingulate gyrus	R	31	13	49	31	5224	3.60	5.39
**Temporal / occipital Lobe**								
Middle temporal gyrus	L	37	-37	-58	1	59513	5.90	12.29
Inferior temporal gyrus	L	20/21	-44	-11	-14	4440	3.96	5.89
Superior / middle temporal gyrus	L	21/38	-39	7	-25	918	3.71	5.11
Middle occipital / temporal gyrus	R	37	37	-61	1	49779	5.50	11.40
**B: Non-smokers: new GWLs < neutral cues**								
**Frontal Lobe**								
Medial / superior frontal gyrus	R	9/10	33	42	28	804	3.38	3.86
Middle frontal gyrus / Precentral gyrus	L	8/9	-36	20	40	121	3.55	4.35
**Occipital Lobe**								
Lingual gyrus / Cuneus	R	17/18	8	-82	2	4362	3.67	4.96

Abbreviations: R: right, L: left; Centre of mass: Talairach Coordinate; Size: cluster size; Ø: mean value

#### New GWLs versus old text-only labels

**Fig 3:**
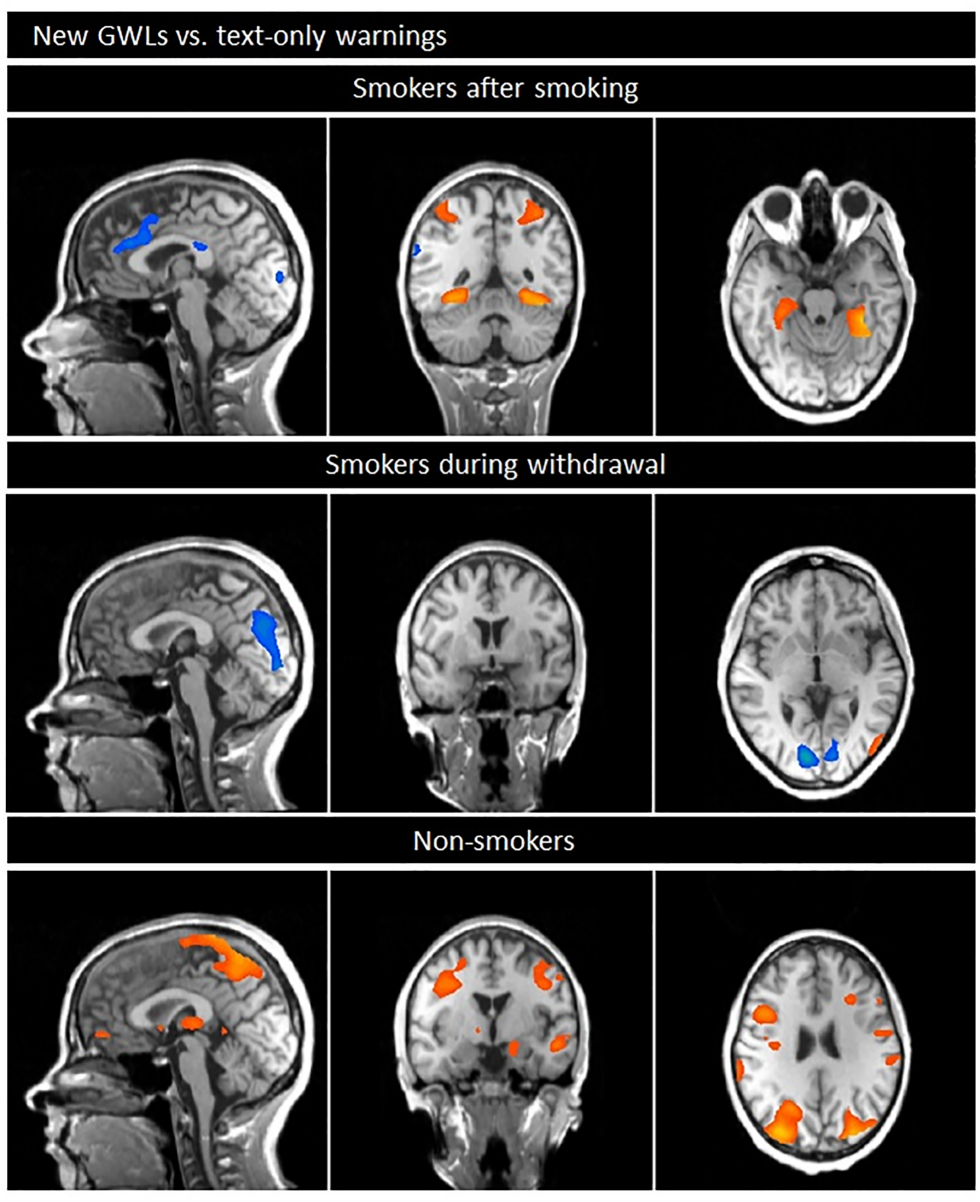
Comparison of neuronal responses during the presentation of the new GWLs minus old text-only labels.

In the comparison of the new GWLs with old text-only labels, *smokers after smoking* showed increased BOLD responses especially in subcortical areas, including the parahippocampal gyrus/fusiform gyrus, globus pallidus) as well as parietal areas (e.g. superior/inferior parietal lobule, precuneus). By contrast, the activation in frontal and temporal brain regions, including the superior, middle and inferior frontal gyrus, the anterior cingulate gyrus and the insula are reduced (see Table 5, Fig 3).

**Table 5:** Smokers after smoking: Comparison of new GWLs and old text-only labels (Fixed effects analysis q(FDR) < 0.01, T: 3.29–8.0, p<0.001020).

			Center of Mass			Size	T-value	
Brain region	Site	BA	x	y	z		Ø	Max
**A: Smokers after smoking: new GWLs > old text-only labels**								
**Subcortical / Occipital Lobe**								
Lingual / fusiform gyrus parahippocampal gyrus	L	18/19	-35	-65	-4	27227	5.30	9.19
Parahippocampal / fusiform gyrus Middle occipital gyrus	R	17/18/19 20/28/35-37	34	-65	-1	24870	4.64	9.20
**Parietal Lobe**								
Precuneus / Postcentral Gyrus Superior / inferior parietal lobule	R	5/7/40	32	-49	54	4276	3.96	5.80
Precuneus / Postcentral Gyrus Superior / inferior parietal lobule	L	7/40	-31	-47	51	2732	3.81	4.99
**B: Smokers after smoking: new GWLs < old text-only labels**								
**Frontal Lobe**								
Cingulate gyrus Medial frontal gyrus	L	6/9/24/32	-5	22	31	2034	3.77	4.66
(Anterior) cingulate gyrus Medial frontal gyrus / Insula	R	9/13/24/32	23	23	20	18921	3.91	5.55
(Posterior) cingulate gyrus	L	23/31	-7	-26	28	1886	3.80	4.83
(Posterior) cingulate gyrus	R	23/31	5	-24	28	577	3.55	3.99
Medial frontal gyrus / ACC	L	9/24/32	-10	37	22	886	3.62	4.48
**Temporal Lobe**								
Superior temporal gyrus Inferior Parietal Lobule	R	13/22/40/42	59	-39	21	833	3.94	5.22
**Subcortical / Occipital Lobe**								
Lingual gyrus / Inferior Occipital Gyrus	R	17/18	9	-85	-3	1882	3.89	4.98
Insula / Precentral gyrus	L	13/44	-35	5	6	9100	4.31	7.64
Insula	R	13	35	13	10	7464	3.92	5.55
Thalamus / Putamen Lentiform nucleus	R		23	-10	8	975	3.64	4.44

Abbreviations: R: right, L: left; Centre of mass: Talairach Coordinate; Size: cluster size; Ø: mean value

*Smokers during withdrawal* demonstrated very small differences between both conditions: the BOLD responses were slightly increased during the presentation of the new GWLs in the parahippocampal gyrus/fusiform gyrus and occipital regions and slightly decreased, predominantly in the cuneus/precuneus as well as the middle/superior frontal gyrus (see Table 6, Fig 3).

**Table 6:** Smokers during withdrawal: Comparison of new GWLs and old text-only labels (Fixed effects analysis q(FDR)< 0.01, T: 3.72–8.0; p < 0.000198).

			Center of Mass			Size	T-value	
Brain region	Site	BA	x	y	z		Ø	Max
**A: Smokers during withdrawal: new GWLs > old text-only labels**								
**Temporal Lobe**								
Parahippocampal gyrus / Fusiform gyrus	R	20/36/37	34	-35	-17	586	4.03	4.59
**Occipital Lobe**								
Middle / inferior occipital Gyrus Inferior temporal Gyrus	L	18/19/37	-47	-72	0	624	4.05	4.54
**B: Smokers during withdrawal: new GWLs < old text-only labels**								
**Frontal Lobe**								
Superior / middle frontal gyrus	L	10	-22	57	7	1097	4.21	5.12
**Occipital Lobe**								
Cuneus / Precuneus	R	17/18/23/30/31	5	-74	18	7575	4.35	6.38
Cuneus / Precuneus	L	17/18/23/30/31	-7	-74	18	2434	4.15	5.04
Lingual gyrus / Cuneus	L	17/18	-12	-77	2	2181	4.07	5.17

Abbreviations: R: right, L: left; Centre of mass: Talairach Coordinate; Size: cluster size; Ø: mean value

*Non-smokers* showed increased BOLD responses during the presentation of the new GWLs compared to text-only labels especially in subcortical areas (e.g. parahippocampal gyrus, amygdala, thalamus, hippocampus, putamen, caudate, globus pallidus), frontal brain regions (including the middle and inferior frontal gyrus), parietal areas (e.g. superior parietal lobule/precuneus), temporal areas (e.g. middle/superior temporal gyrus) and occipital areas (e.g. cuneus/precuneus, superior/inferior occipital gyrus). Responses especially in the lingual gyrus/inferior occipital gyrus were decreased (see Table 7, Fig 3).

**Table 7:** Non-smokers: Comparison of new GWLs and old text-only labels (Fixed effects analysis q(FDR)< 0.01, T: 3.08–8.0; p < 0.002090).

			Center of Mass			Size	T-value	
Brain region	Site	BA	x	y	z		Ø	Max
**A: Non-smokers: new GWLs > old text-only labels**								
**Subcortical / Occipital Lobe**								
Lingual gyrus / Cuneus / Precuneus	R	18/19/31	33	-63	4	56842	5.42	9.33
Lingual gyrus / Cuneus / Precuneus Superior occipital gyrus	L	18/19/31	-32	-25	-13	56794	5.79	11.15
Thalamus	R		10	-21	8	6582	3.71	5.66
Thalamus	L		-15	-27	7	6046	3.79	5.34
Amygdala / Parahippocampal gyrus	L	28/34	-17	-7	-12	717	3.38	4.04
Hippocampus / Parahippocampal gyrus	L	36	-32	-25	-13	611	3.60	5.35
Caudate / Lentiform nucleus Globus pallidus / Putamen	R		9	5	6	1660	3.55	4.66
**Temporal Lobe**								
Superior / middle temporal gyrus	L	21/22/38	-53	-8	-8	1276	3.53	4.74
Amygdala / Superior temporal gyrus	R	13/21/22/38	45	3	-9	479	3.31	3.96
**Frontal Lobe**								
Inferior / medial frontal gyrus	L	13/7	-33	29	-3	1047	4.03	6.07
Inferior / middle frontal gyrus	L	9/45/46	-48	24	18	897	3.09	3.35
Precentral gyrus / Middle frontal gyrus	L	3/4/6	-38	-11	-50	4971	3.60	5.20
Middle frontal gyrus / Precentral gyrus	R	6	32	-8	42	5661	3.54	4.73
Post-/precentral gyrus	R	1–4	27	-30	66	565	3.32	3.77
**Parietal Lobe**								
Precuneus / Superior parietal lobule	R	7	19	-54	48	30739	4.10	6.87
Precuneus / Superior parietal lobule	L	7	-23	-51	50	39127	4.41	6.69
**B: Non-smokers: new GWLs < old text-only labels**								
**Occipital Lobe**								
Lingual gyrus / Inferior Occipital Gyrus Cuneus	R	17/18	10	-88	-5	2373	3.97	5.58

Abbreviations: R: right, L: left; Centre of mass: Talairach Coordinate; Size: cluster size; Ø: mean value

#### Smoking urge and aggression

Craving increased in the group of smokers after smoking after the fMRI session compared to before (*T* (22) = -2.65, *p* = .015; Table 8). Difference between pre and post fMRI for the intention to smoke in all smokers was not significant, nor for craving in smokers during withdrawal (*p* > .185). The level of aggression did not change after the fMRI session for any group (*p* > .536).

**Table 8:** Results of the QSU in smokers and non-smokers before and after the acquisition of the fMRI data.

	Before fMRI session				After fMRI session			
	Intention to smoke		Craving		Intention to smoke		Craving	
	M	SD	M	SD	M	SD	M	SD
Non-smokers	12.4	2.62	10.0	0.00	11.7	2.91	10.0	0.00
Smokers after smoking	52.6	15.06	19.2	7.17	57.3	15.66	24.6	11.26
Smokers during withdrawal	64.7	16.35	32.4	13.30	63.9	16.54	34.6	17.03

Abbreviations: M: mean value; SD: standard deviation

The results also showed that craving and intention to smoke were significantly higher for smokers during withdrawal than for smokers after smoking before the fMRI session (*T* < -2.65, *p* < .015). After the fMRI session, craving was also significantly higher in smokers during withdrawal than in smokers after smoking (*T* (21) = -2.11, *p* = .047). Aggression scores did not differ significantly between smokers after smoking and smokers during withdrawal, neither before nor after the fMRI session (|*ts*| < 1.70, p > .103).

## Discussion

The present study investigated the neuronal responses to the new German GWLs in non-smokers and smokers. We focused especially on the effect of smoking status on neurobiological responses comparing smokers after smoking and smokers during withdrawal. While undergoing fMRI, new GWLs, old text-only warnings and neutral pictures were presented to the participants. To our knowledge the effect of smoking status on BOLD responses has not been considered so far: up to now, neuronal responses of smokers during withdrawal have not been examined (e.g. Newman-Norlund et al., 2014; Wang et al., 2015).

### New GWLs vs neutral pictures

The presentation of the new GWLs warnings to *smokers after smoking* resulted in increased activations especially in subcortical, temporal and frontal brain regions including amygdala, hippocampus, caudate, thalamus, medial frontal regions and the DLPFC compared to neutral pictures. These regions are especially associated with emotional processing (e.g. amygdala, hippocampus, parahippocampal gyrus, insula) as well as cognitive processes (e.g. medial/prefrontal cortex/DLPFC) including attention and working memory. The insula is often related to a network that includes the amygdala and the prefrontal cortex and is important for the conversion of sensory information into emotions [25,47]. In addition, the importance of the insula for the perception of hazards of smoking has been demonstrated [48]. In addition, brain regions which are thought to be associated with visual processing (e.g. occipital cortex, precuneus) and earlier stages of processing of emotional stimuli (e.g. fusiform cortex) [49] seem to be involved.

*Smokers during withdrawal* also showed increased responses predominantly in frontal, temporal/occipital and subcortical areas (e.g. amygdala/hippocampus, inferior/middle frontal gyrus, middle/inferior temporal gyrus); however, the differences between GWLs and neutral pictures were smaller than in smokers after smoking. By contrast, the findings of non-smokers were comparable to those of smokers after smoking: areas of emotional processing, areas of visual processing and areas of cognitive processing showed pronounced BOLD responses during the presentation of GWLs.

The findings that GWLs activate brain regions that are involved in cognition, emotion and memory formation are consistent with current literature [25,50]: these studies demonstrated the importance of amygdala/hippocampus, insula and visual association cortices. The neural activity in regions which are involved in emotional and cognitive processing of warning messages is a predictor of positive cessation outcomes [32,51]. Numerous neuroscientific studies confirmed the prominent role of the amygdala in emotional processing in a number of sensory modalities [30,31,52] especially threat and fear [53,54]. Particularly high emotional salient GWLs were associated with activations in the amygdala and the hippocampus [25,27], probably indicating higher emotional responses as well as enhanced memory. The insula has shown to be another important brain region during the presentation of GWLs. This area is especially linked to disgust, e.g. induced by mutilation and contamination [55,56,57]. Interestingly the differences between neuronal responses to GWLs and neutral pictures were weaker in smokers after deprivation than in smokers after smoking, and in non-smokers. This may indicate that the effect of the new warnings labels on important brain areas of smokers is strongest in a limited period of time. After a certain time of deprivation, smokers seem to be less affected by the pictures.

### New GWLs vs text-only labels

Former studies have already demonstrated enhanced emotional responses to pictorial warning labels compared to text-only warnings [9] as well as beneficial effects of GWLs on behaviour correlates. However, neurobiological aspects that may underly this difference have not been considered so far.

In the present study the comparison of neurobiological correlates of the new GWLs with the old text-only labels demonstrated in particular the importance of affective brain regions in *smokers after smoking* and in *non-smokers*: the differences between categories (new GWLs vs. text-only labels) were smaller than regarding the comparison of GWLs and neutral pictures.

Subcortical areas and occipital regions (e.g. parahippocampal gyrus, fusiform gyrus, lingual gyrus) as well as regions of the visual association cortex were predominantly activated in *smokers after smoking* during the presentation of new GWLs. By contrast, text-only labels were related to responses, e.g. in the posterior and anterior cingulate gyrus, medial frontal gyrus, insula/precentral gyrus. These responses may indicate enhanced higher cognitive processes, including working memory and response inhibition.

In *non-smokers* emotion-relevant areas (e.g. amygdala, parahippocampal areas, hippocampus) as well as areas related to cognitive processes (e.g. frontal regions) and brain regions involved in the transmission of sensory signals to the cerebral cortex (e.g. thalamus) were stronger linked to the presentation of GWLs than to text-only labels.

By contrast, *during withdrawal*, the neural responses during the presentation of GWLs and text-only labels differed only marginally: new GWLs were related to increased responses, e.g. in the parahippocampal area / lingual gyrus and the occipital gyrus. Text-only labels, by contrast, led to enhanced BOLD responses, e.g. in the cuneus/precuneus. Comparable responses are often seen during resting-state tasks.

Overall the results may lead to the presumption that new GWLs images are more emotional salient than textual information and lead to enhanced neuronal responses e.g. in brain regions that are related to the emotion network in *non-smokers* and *smokers after smoking*. In *smokers during withdrawal*, warning pictures seem to have little effect. One reason for the slight response in this group could be that craving for the drug is induced by mentioning cigarettes/smoking instead of considering the message in the warning.

One drawback of the comparison of new GWLs and old text-only warning is that these information do not only differ in their emotional intensity but also the use of verbal (text-only warnings) as compared to the combination of visual and verbal information (new GWLs).

Former studies have reported activation especially of the ventral anterior cingulate cortex including the subgenual cortex [58,59], in the amygdala [59,60] as well as the posterior cingulate cortex, the inferior and superior frontal cortex, the inferior and middle temporal gyrus and the thalamus [61] in response to emotionally salient words. The presentation of negative pictures is related to activations especially in the amygdala, the ventral striatum, the insula, the anterior cingulate cortex, the medial prefrontal cortex and the orbitofrontal cortex [62,63,64]. The direct comparison regarding neuronal responses between different modalities indicated that both pictures and words elicited emotional responses with no general superiority for either stimulus modality. However, emotional responses to pictures are modulated by perceptual stimulus features, such as picture complexity [65]. This may indicate that the neurobiological differences may be influenced by the stimulus complexity.

### Behaviour level

With regard to smoking urge, groups differed significantly both in the intention to smoke and in craving before the fMRI session, assumable due to the 12 hours of withdrawal in the condition smokers during withdrawal. Smokers during withdrawal didn’t show any changes in the factor craving after the fMRI which is probably due to an already very high score before the task. This is in accordance with the findings on the neuronal level that smokers in a long period of withdrawal don’t experience any effect of the presented pictures.

Noticeably the group of smokers after smoking showed an increase in craving after the fMRI session. At first, this might seem surprising since the GWLs are supposed to decrease the urge to smoke. One possible explanation for this is that after over 60 minutes of deprivation (questionnaires and fMRI tasks) and additional confrontation of smoking cues, the craving for cigarettes rises naturally.

### Limitations

One limitation of the study is the absence of any behavioural responses during fMRI data acquisition. The lack of behavioural responses makes it impossible to compare neuronal responses with behavioural data. In addition, it leads to an uncertainty over the mental processes of participants during the fMRI session. Apart from this it was not possible to ensure that each participant’s attention was spent on viewing the pictures.

We decided to do not integrate a behavioural task in order to prevent participants to use this task in order to distract themselves from the emotional content of the pictures and associated emotional responses.

Another limitation of the findings results from the experimental design: the findings are limited to smokers that are able to withdraw from smoking for at least ten hours. We are not able to make any statements about smokers where this is not possible.

The differences regarding text-only and new GWL warings may be influenced by the use of verbal and combined verbal and visual information as well as stimulus complexity. These aspects should be carefully be taken into account in further studies.

## Conclusions

The present study examined neural responses to GWL stimuli compared to text-only warnings and neutral pictures in smokers after smoking, smokers during withdrawal and non-smokers. GWLs elicited pronounced activations in a network of brain regions, including the visual association cortex (higher processing of visual information) and emotion-relevant regions including amygdala and insula). Functional differences between picture categories were predominantly present in smokers after smoking and in non-smokers. Smokers during withdrawal demonstrated only small differences between GWLs and neutral/text-only pictures. These results may indicate that the effect of GWLs on cognitive and emotional brain areas is more pronounced after smoking than during withdrawal. During withdrawal, more unspecific processes seem to be important, including the projection of sensory signals to the cerebral cortex and resting-state processes. Neuronal findings were in accordance with behavioural results: in smokers during withdrawal craving was not influenced by the experimental design. The increased craving in smokers after smoking might be related to the duration of non-smoking during the experimental setting.

Altogether, the results suggest that emotional and cognitive reactions to GWLs are predominantly seen in smokers after smoking and in non-smokers. The impact of these pictures during withdrawal seems to be less pronounced.
